# *Trichoderma koningiopsis* (Hypocreaceae) has the smallest mitogenome of the genus *Trichoderma*

**DOI:** 10.3389/fmicb.2023.1141087

**Published:** 2023-06-13

**Authors:** María Lorena Castrillo, Gustavo Ángel Bich, Natalia Soledad Amerio, Marcela Paola Barengo, Pedro Darío Zapata, Mario Carlos Nazareno Saparrat, Laura Lidia Villalba

**Affiliations:** ^1^Laboratorio de Biotecnología Molecular, Instituto de Biotecnología Misiones “Dra. María Ebe Reca”-InBioMis, Universidad Nacional de Misiones, Posadas, Misiones, Argentina; ^2^Consejo Nacional de Investigaciones Científicas y Técnicas (CONICET), Buenos Aires, Argentina; ^3^Facultad de Ciencias Agrarias y Forestales, Instituto de Fisiología Vegetal, Universidad Nacional de La Plata, La Plata, Buenos Aires, Argentina; ^4^Facultad de Ciencias Naturales y Museo, Instituto de Botánica Carlos Spegazzini, Universidad Nacional de La Plata, La Plata, Buenos Aires, Argentina; ^5^Cátedra de Microbiología Agrícola, Facultad de Ciencias Agrarias y Forestales, Universidad Nacional de La Plata, La Plata, Buenos Aires, Argentina

**Keywords:** biocontrol agent, mitochondrial, genome, next generation sequencing, phylogenetic relationships

## Abstract

**Introduction:**

Fungal mitogenomes exhibit remarkable variation in conformation, size, gene content, arrangement and expression, including their intergenic spacers and introns.

**Methods:**

The complete mitochondrial genome sequence of the mycoparasitic fungus *Trichoderma koningiopsis* was determined using the Illumina next-generation sequencing technology. We used data from our recent Illumina NGS-based project of *T. koningiopsis* genome sequencing to study its mitochondrial genome. The mitogenome was assembled, annotated, and compared with other fungal mitogenomes.

**Results:**

*T. koningiopsis* strain POS7 mitogenome is a circular molecule of 27,560 bp long with a GC content of 27.80%. It harbors the whole complement of the 14 conserved mitochondrial protein-coding genes (PCG) such as *atp*6, *atp*8, *atp*9, *cox*1, *cox*2, *cox*3, *cob*, *nad*1, *nad*2, *nad*3, *nad*4, *nad*4L, *nad*5, and *nad*6, also found in the same gene order to other Hypocreales. The mitogenome also contains 26 transfer RNA genes (tRNAs), 5 of them with more than one copy. Other genes also present in the assembled mitochondrial genome are a small rRNA subunit and a large rRNA subunit containing ribosomal protein S3 gene. Despite the small genome size, two introns were detected in the *T. koningiopsis* POS7 mitogenome, one of them in *cox*3 gene and the other in *rnl* gene, accounting 7.34% of this mitogenome with a total size of 2,024 bp. A phylogenetic analysis was done using the 14 PCGs genes of *T. koningiopsis* strain POS7 mitogenome to compare them with those from other fungi of the Subphyla Pezizomycotina and Saccharomycotina. *T. koningiopsis* strain POS7 was clustered together with other representatives of *Trichoderma* lineage, within the Hypocreales group, which is also supported by previous phylogenetic studies based on nuclear markers.

**Discussion:**

The mitochondrial genome of *T. koningiopsis* POS7 will allow further investigations into the taxonomy, phylogenetics, conservation genetics, and evolutionary biology of this important genus as well as other closely related species.

## 1. Introduction

Mitochondria use electron transport coupled with oxidative phosphorylation to generate ATP and these organelles also participate in several other cellular important functions (Burger et al., 2003). Mitochondrial DNA or mitochondrial genome, also known as mitogenome, is made up of genes that encodes a small number of proteins, whose mRNAs are translated by a distinctive mitochondrial protein-synthesizing system (Burger et al., 2003; Kwak, 2020). Evolutionarily, mitogenomes exhibit remarkable variation in conformation, size, actual gene content, arrangement and expression, including their intergenic spacers and introns (Gray et al., 1999; Guha et al., 2018; Li et al., 2019). Within the fungi mitogenome can exist as either linear or circular molecules (Hausner, 2003; Medina et al., 2020).

Many fungi show similar morphological features, which are broadly dependent upon culture conditions, and so pleomorphic, which usually difficult their identification using only traditional morphological characters. Therefore, a fungal natural classification must be including phylogenetic tools. However, these taxonomic analyses of fungi using phylogenetic inferences need to be combined with several molecular markers, such as those from the mitogenome, which can be a good complement to explain their variation (Chen et al., 2019).

Mitochondria contain their own genome, and they may provide adequate information for phylogenetic studies based on their comparisons with ones from other reference taxa (Ghikas et al., 2006; Kanzi et al., 2016). However, fungal mitogenomes have been less studied than their animal or plant counterparts, limiting a complete understanding of their genetic characteristics and evolutionary histories (Aguileta et al., 2014).

Recently, development of high-throughput sequencing technologies and related bioinformatics tools have allowed considerably greater numbers of nucleotides to be characterized and the genome-scale data to be more easily assembled. Compared to the whole-genome data, mitogenomes are more readily sequenced with broader taxon sampling and a reasonable cost (Song et al., 2020). The number of complete filamentous fungal mitogenome sequences has significantly increased in the last years facilitating evolutionary and systematic studies (Mardanov et al., 2014; Megarioti and Kouvelis, 2020; Fatma et al., 2023). Fungal mitogenomes typically contain 15 standard protein-coding genes, two rRNA genes and a variable number of tRNA genes. These protein-coding genes are *atp*6, *atp*8, *atp*9, *cob*, *cox*1, *cox*2, *cox*3, *nad*1, *nad*2, *nad*3, *nad*4, *nad*4L, *nad*5, *nad*6, and *rps*3 (Lang, 2018), being some of them absent in certain fungal mitogenomes (Zhang and Zhang, 2019).

The independent inheritance from nuclear genomes, a high copy number, and several available molecular markers has made the mitogenomes widely used as a tool for studying evolution, phylogenetics, population genetics, and comparative or evolutionary genomics for some fungi (Van de Sande, 2012; Chen et al., 2019; Li et al., 2020; Wang et al., 2020).

Fungi belonging to the *Trichoderma* genus (Hypocreaceae, Hypocreales, Sordariomycetes, Pezizomycotina, Ascomycota) are ubiquitous inhabitants in different habitats, being dominate in a broad spectrum of soils and/or decaying organic matter. Species of this genus are economically important, in part because of their mycoparasitic ability, but also as they can stimulate plant resistance, and plant growth and development which increase crop production. Among these uses, *T. koningiopsis* was reported as a fungal species that can be used in biocontrol and other biotechnological applications (Samuels et al., 2006; Castrillo et al., 2015; Amerio et al., 2020; Castrillo et al., 2021a).

*Trichoderma* whole genome analyses have been reported previously (Halliwell and Griffin, 1973; Antal et al., 2002; Chambergo et al., 2002; Martinez et al., 2008; Kubicek et al., 2011; Studholme et al., 2013; Steindorff et al., 2014; Xie et al., 2014; Baroncelli et al., 2015, 2016; Shi-Kunne et al., 2015; Castrillo et al., 2017; Proctor et al., 2018). However, in spite of the extensive studies on biocontrol and nuclear genes of *Trichoderma* genus, information about its mitogenomes remains largely unknown. To date, even if *Trichoderma* genus has more than 300 known species, only 11 mitogenomes are available, including *Trichoderma arundinaceum*, *Trichoderma asperellum*, *Trichoderma atroviride*, *Trichoderma cornu-damae*, *Trichoderma gamsii*, *Trichoderma hamatum*, *Trichoderma harzianum*, *Trichoderma pseudokoningii*, *Trichoderma reesei*, *Trichoderma simmonsii* and *Trichoderma virens* (Kwak, 2020; Cai and Druzhinina, 2021). The characteristics of mitogenomes belonging to different representatives of *Trichoderma* genus are needed to elucidate the understanding of the evolution and potential technological use of specific isolates of this genus. The objectives of this work were to determine the complete mitogenome sequence of the *T. koningiopsis* POS7 strain and to compare its mitogenome organization with that of other known mitogenome from Subphylum Pezizomycotina.

## 2. Materials and methods

### 2.1. Strain

*Trichoderma koningiopsis* strain POS7 (Hypocreaceae) was isolated from soil samples (Posadas, Misiones, Argentina; Castrillo et al., 2017). *Trichoderma koningiopsis* POS7 was sequenced, NCBI ID: 337941, Bioproject PRJNA356137, Biosample SAMN06106985, WGS MRBD 00000000, ITS reference sequence KT030879, and was deposited at the Universidad Nacional de Misiones under accession number LBM116.

### 2.2. Mitochondrial genome assembly

In this study, the mitogenome of *T. koningiopsis* POS7 was determined from HiSeq Illumina reads data from whole genome sequencing of *T. koningiopsis* POS7 were used (Castrillo et al., 2017). All reads (7,773,936 reads) were used to map and assemble by comparing with mitochondrial DNA of other species of *Trichoderma* using the software Geneious 9.1.8 (Kearse et al., 2012). The reported mitogenomes of *T. gamsii* and *T. asperellum* (NC_030218 and NC_037075, respectively) were used as reference to map reads and assemble the mitogenome of *T. koningiopsis*. A total of 88,833 reads were retrieved from the complete genome sequencing project to assemble the mitogenome sequence. The contigs were manually curated into the final single genomic sequence. A single mitochondrial contig was extracted from the assembly for further analyses.

### 2.3. Mitochondrial genome annotation

Initial open reading frames were identified using ORF finder^1^ using the mold mitochondrial genetic code and Geneious 9.1.8 program. The protein-coding genes, rRNA, and tRNA genes of *T. koningiopsis* were predicted and curated manually based on comparisons with previously published *Trichoderma* mitogenome.

This initial annotation was helped using MFannot online tool^2^ based on Genetic Code 4 (Molds). The tRNAs genes were also identified using tRNAscan-SE 2.0 (Chan and Lowe, 2019). Putative functional assignments of genes were made based on sequence similarity to characterize fungal mitochondrial genes using BLASTN searches against NCBI databases (Altschul et al., 1990). Then manual examination was performed to rectify possible annotation errors. After confirming the presence of all the conserved mitochondrial genes in the assembled contig, the displayed overlaps at both ends were used to circularize the mitogenome.

Codon usage was determined using the online Sequence Manipulation Suite software (Stothard, 2000), and using the genetic code 4 for mold mitochondrial. The physical map of *T. koningiopsis* POS7 mitogenome was generated using the same software Geneious 9.1.8.

### 2.4. Phylogenetic analyses

Phylogenetic analyses were performed using the 14 conserved mitochondrial genes of a database constructed with selected taxa from the Subphylum Pezizomycotina (Cardona et al., 2018). In addition, one species belonging to the Subphylum Saccharomycotina, *Wickerhamomyces canadensis* was used as outgroups.

The complete mitogenomes of 22 species of Hypocreales and Sordariales order available in GenBank were retrieved, and two of them were annotated in this study (Table 1). The sequences of 14 conserved protein genes namely *atp*6, *atp*8, *atp*9, *cox*1, *cox*2, *cox*3, *cob*, *nad*1, *nad*2, *nad*3, *nad*4, *nad*4L, *nad*5, and *nad*6 from each species were concatenated and used for phylogenetic analyses.

**Table 1:** Species included in the phylogenetic analysis.

Genbank accession number	Species	Class	Order	Mitogenome size (bp)	Reference
NC_023268	*Acremonium chrysogenum*	Sordariomycetes	Hypocreales	27,266	Jelen et al. (2016)
NC_022835	*Metacordyceps chlamydosporia*	Sordariomycetes	Hypocreales	25,615	Jelen et al. (2016)
NC_017930	*Fusarium oxysporum*	Sordariomycetes	Hypocreales	34,477	Jelen et al. (2016)
NC_008248	*Verticillium dahlia*	Sordariomycetes	Glomerellales	27,184	Jelen et al. (2016)
KR704425	*Verticillium nonalfalfae*	Sordariomycetes	Glomerellales	26,139	Jelen et al. (2016)
NC_008068	*Metarhizium anisopliae*	Sordariomycetes	Hypocreales	24,673	Jelen et al. (2016)
NC_004514	*Lecanicillium muscarium*	Sordariomycetes	Hypocreales	24,499	Jelen et al. (2016)
NC_001329	*Podospora anserine*	Dothideomycetes	Mycosphaerellales	100,314	Jelen et al. (2016)
KC683708	*Neurospora crassa*	Sordariomycetes	Sordariales	64,840	Jelen et al. (2016)
D31785	*Wickerhamomyces canadensis*	Saccharomycetes	Saccharomycetales	27,694	Ghikas et al. (2006)
MH211586	*Beauveria bassiana*	Sordariomycetes	Hypocreales	31,258	Genbank
NC_037075	*Trichoderma asperellum*	Sordariomycetes	Hypocreales	29,999	Kwak (2020)
MN125601	*Trichoderma atroviride*	Sordariomycetes	Hypocreales	32,758	Kwak (2020)
NC_030218	*Trichoderma gamsii*	Sordariomycetes	Hypocreales	29,303	Kwak (2020)
MF287973	*Trichoderma hamatum*	Sordariomycetes	Hypocreales	32,763	Kwak (2020)
NC_003388	*Trichoderma reesei*	Sordariomycetes	Hypocreales	42,130	Kwak (2020)
MN564945	*Trichoderma harzianum*	Sordariomycetes	Hypocreales	27,632	Genbank
NC_052832	*Trichoderma lixii*	Sordariomycetes	Hypocreales	29,791	Genbank
NC_063562	*Trichoderma simmonsii*	Sordariomycetes	Hypocreales	28,668	Genbank
CP071122	*Trichoderma virens*	Sordariomycetes	Hypocreales	31,081	Genbank
MW525445	*Trichoderma cornudamae*	Sordariomycetes	Hypocreales	94,608	Genbank
NC_065768	*Trichoderma afroharzianum*	Sordariomycetes	Hypocreales	29,517	Genbank
OW971927	*Trichoderma pseudokoningii*	Sordariomycetes	Hypocreales	45,112	Genbank
MT816499	*Trichoderma koningiopsis*	Sordariomycetes	Hypocreales	27,560	This work

Sequence alignment was performed using the Clustal W program of the MEGA 11package (Tamura et al., 2021) with parameters set to default. Phylogenetic reconstruction were inferred using the Maximum Likelihood (ML) and Neighbor Joining (NJ) methods using the Mega 11 package (Tamura et al., 2021) with parameters set to default. Boostrap values were determined using 1,000 replicates.

## 3. Results and discussion

### 3.1. General features of *T. koningiopsis* POS7 mitochondrial genome

The rapid development of next generation sequencing technologies has resulted in increasing the numbers of available mitogenomes from various organisms (Brankovics et al., 2017; Li et al., 2019). Fungal mitogenomes are among the most variable ones compared to others from several eukaryotic groups, both in size and organization (Hausner, 2003; Formey et al., 2012; Li et al., 2019; Lee et al., 2022).

So far, 2028 fungal mitochondrion NCBI Reference Sequences (RefSeq) are available in NCBI Database (https://www.ncbi.nlm.nih.gov/nuccore, January 2023). The overall average size of fungal mitogenomes sequenced is 50,512 bp (Jelen et al., 2016), though it can vary greatly, ranging from 11 kb in *Hanseniaspora uvarum* (Saccharomycetales, Pramateftaki et al., 2006; Megarioti and Kouvelis, 2020) to 236 kp in *Rhizoctonia solani* (Cantharellales from phylum Basidiomycota) or 272 kb in *Morchella importuna* (Pezizales, James et al., 2013; Losada et al., 2014; Li et al., 2020).

In this study, the mitogenome of the mycoparasitic fungus *T. koningiopsis* POS7 had a total size of 27,560 bp. The mtDNA sequence mean coverage in the assembly was 326X. This newly sequenced mitogenome was deposited in the GenBank database with the accession number MT816499. The size of *T. koningiopsis* POS7 mitogenome was the smallest genome among the 13 mitogenomes available in the *Trichoderma* genus (Table 1).

The GC content in organisms is considered to be influenced by mutational bias, selection, and biased recombination related to DNA repair, which could provide useful information regarding the evolution of different species (Li et al., 2019). The GC content of the *T. koningiopsis* POS7 mitogenome was 27.8%. This is in concordance with the other known *Trichoderma* mitogenomes evidencing no particular skew, ranging from 26.4% in *T. reesei* (NC_003388) to 28% in *T. asperellum* (NC_037075). These findings indicate that *Trichoderma* species may ultimately be subject to similar selection pressures; nevertheless, deeper studies with additional genomic regions are needed to confirm this.

Forty-three genes were detected into *T. koningiopsis* POS7 mitogenome, all of them being located on the sense strand (Table 2). The latter is in accordance with most Ascomycota mitogenomes where all genes are transcribed from the same DNA strand (Ghikas et al., 2006). However, in some Ascomycota some genes are located on the antisense strand as in *Verticillium nonalfalfae* (Glomerellales, Haridas and Gantt, 2010; Costa et al., 2016; Jelen et al., 2016).

**Table 2:** Gene features of the *Trichoderma koningiopsis* POS7 mitochondrial genome.

Gene	Start position	Stop position	Length	Strand	Start codon	Stop codon
*cox*1	68	1,660	1,593	+	AUG	UAA
*cox*2	21,428	22,177	750	+	AUG	UAA
*cox*3	8,900	9,983	1,084	+	AUG	UAA
*Cob*	25,811	26,980	1,170	+	AUG	UAA
*nad*1	2,238	3,395	1,158	+	AUG	UAA
*nad*2	18,761	20,428	1,668	+	AUG	UAA
*nad*3	20,429	20,842	414	+	AUG	UAA
*nad*4	3,537	4,994	1,458	+	AUG	UAA
*nad*4L	22,485	22,754	270	+	AUG	UAA
*nad*5	22,854	24,920	2,067	+	AUG	UAA
*nad*6	10,228	10,980	753	+	AUG	UAA
*atp*6	5,764	6,546	783	+	AUG	UAA
*atp*8	5,434	5,580	147	+	AUG	UAA
*atp*9	21,038	21,262	225	+	AUG	UAA
*rps*3	14,393	15,793	1,401	+	AUU	UAA
*rns*	6,899	8,401	1,503	+	–	–
*rnl*	11,758	14,172	2,415	+	–	–
tRNA genes for	Start position	Stop position	Length	Strand	Anticodon	
Arg	1,793	1,863	71	+	TCT	
Phe	5,125	5,195	71	+	AAA	
Tyr	8,445	8,528	84	+	GTA	
Asp	8,615	8,688	74	+	GTC	
Ser	8,694	8,777	84	+		
Asn	8,782	8,853	72	+	GTT	
Gly	10,037	10,107	71	+	TCC	
Val	10,898	10,969	72	+	TAC	
Ile	11,130	11,201	72	+		
Ser	11,277	11,363	87	+	TGA	
Trp	11,372	11,443	72	+	TCA	
Pro	11,497	11,568	72	+		
Thr	16,467	16,537	71	+		
Glu	16,543	16,615	73	+	TTC	
Met	16,616	16,686	71	+	CAT	
Met	16,826	16,898	73	+	CAT	
Leu	16,903	16,985	83	+	TAA	
Ala	17,067	17,138	72	+	TGC	
Phe	17,144	17,216	73	+	GAA	
Lys	17,217	17,289	73	+	TTT	
Leu	17,686	17,768	83	+		
Gln	18,066	18,138	73	+		
His	18,323	18,396	73	+	GTG	
Met	18,565	18,636	72	+	CAT	
Arg	22,276	22,346	71	+		
Cys	27,033	27,106	74	+		

**Figure 1:**
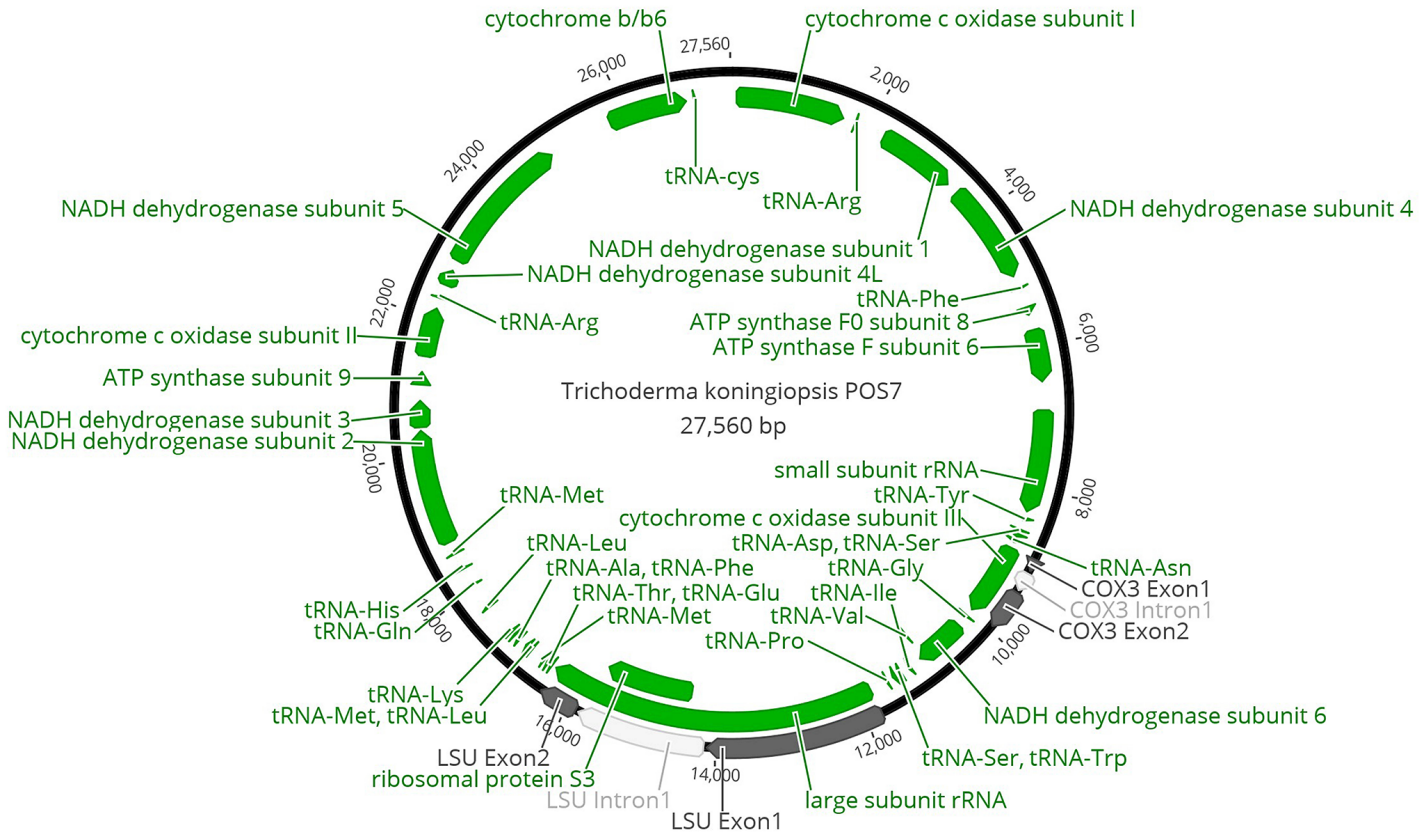
Circular map of *Trichoderma koningiopsis* POS7 mitochondrion. Proteins, rRNA and tRNA genes were indicated in green. Exons were indicated in dark grey and introns were indicated in white.

The entire genome found in the mitochondrion of *T. koningiopsis* POS7 after its curated annotations is shown in Figure 1.

### 3.2. Protein-coding genes and codon usage

The *T. koningiopsis* POS7 mitogenome had the conserved set of 14 mitochondrial protein-coding genes: the ATP synthase subunits (*atp*6, *atp*8, and *atp*9), the cytochrome oxidase subunits (*cox*1, *cox*2, and *cox*3), the reduced nicotinamide adenine dinucleotide ubiquinone oxidoreductase subunits (*nad*1, *nad*2, *nad*3, *nad*4, *nad*4L, *nad*5, and *nad*6), and the apocytochrome b (*cob*). All these genes were located in the same order in the other species of *Trichoderma* genus (Supplementary Table S1). Remarkably, in the *T. koningiopsis* mitogenome, inside the *cox*3 gene an intron of 274 bp was located. Similarly, the mitogenomes of *T. hamatum* (MF287973) and *T. asperellum* (NC_037075) had an intron located also into the *cox*3 gene (Table 3).

**Table 3:** Features of *Trichoderma* mitochondrial genomes that were used for comparative analyses.

Strain	Genbank accession no.	Mitochondrial genome size (bp)	AT content (%)	GC content (%)	rRNA genes	tRNA genes	Intergenic regions (%)	Numbers of introns	Located of introns	Introns (%)
*Trichoderma asperellum*	NC_037075	29,999	72	28	1	25	29.44	1	**27894.29011/COX III**	3.73
*Trichoderma atroviride*	MN125601	32,758	72.4	27.6	2	27	24.63	3	4005.5061/cytochrome b 5380.5834/cytochrome b**7863.9141/COX I**	8.52
*Trichoderma gamsii*	NC_030218	29,303	72.4	27.6	2	26	25.03	0		0
*Trichoderma hamatum*	MF287973	32,763	72.3	27.7	2	26	25.03	4	**6054.7165/COX III 19746.21068/COX II 21220.22338/COX II 29198.22338/COX I**	14.73
*Trichoderma reesei*	NC_003388	42,130	73.6	26.4	2	23	17.74	9	12161.13696/LSU rRNA**22961.23212/COX II** 27776.29089/cytochrome b 29197.30256/cytochrome b**32006.33275/COX I 33453.34662/COX I 34948.35983/COX I 36287.37512/COX I 37705.39764/COX I**	26.02
*Trichoderma harzianum*	MN564945	27,632	72.4	27.6	2	24	20.76	0		0
*Trichoderma lixii*	NC_052832	29,791	72.6	27.4	2	25	35.65	2	11375.13009/S3 18580.19660/ATP 9	9.11
*Trichoderma simmonsii*	NC_063562	28,668	72.4	27.6	2	26	27.38	0		0
*Trichoderma virens*	CP071122	31,081	72.4	27.6	2	25	19.24	3	11217.12736/LSU rRNA 24011.24636/cytochrome b 24692.25265/cytochrome b	8.75
*Trichoderma cornu-damae*	MW525445	94,608	72.1	27.9	2	24	26.83	25	14302.16410/LSU rRNA 16672.18123/Lsu rRNA 18700.20380/LSU rRNA 20778.23006/LSU rRNA 38429.39809/NAD 240197.41569/NAD 241996.43372/NAD 2**47120.48457/COX II 48605.49723/COX II 49853.54018/COX II 54308.56280/COX II** 60099.61523/NADH 4 L 61803.63639/NADH 566635.69594/cytochrome b 69898.70949/cytochrome b 71269.73349/cytochrome b**74604.77204/COX I 77274.78525/COX I 78631.79917/COX I 80242.81649/COX I 81672.82713/COX I 83038.84305/COX I 84531.87395/COX I** 85795.87165/orf447	48.12
*Trichoderma afroharzianum*	NC_065768	29,517	72.3	27.7	2	22	18.75	4	5201.6445/COX I 18993.19887/LSU rRNA 20768.22408/LSU rRNA 27635.27856/LAGLIDADG	13.55
*Trichoderma pseudokoningii*	OW971927	45,112	72.6	27.4	2	24	26.26	8	11945.1348/LSU rRNA**24493.24883/COX II** 29252.29647/cytochrome b 31020.32079/cytochrome b**33670.34861/COX I 35039.35112/COX I 37723.38948/COX I 39141.41198/COX I**	19.52
*Trichoderma koningiopsis*	MT816499	27,560	72.2	27.8	2	26	21.63	2	**9119.9392/COX III** 14145.15894/LSU rRNA	7.34

In bold values were the introns in cox genes (coxI, coxII and coxIII).

Codon usage analyses indicated that the most frequently used codons in *T. koningiopsis* POS7 mitogenome were ATT (for isoleucine; Ile), TTT (for phenylalanine; Phe), and ATA (for isoleucine; Ile). The high frequency of AT content of these codons (72.2%) is in agreement to the high AT content of the other12 *Trichoderma* analyzed mitogenomes [from 72.1% in *T. cornu-damae* (MW525445) to 73.6% in *T. reesei* (NC_003388)].

Genes were also analyzed with respect to their start and stop codons. Among the predicted genes, the “AUG” initiation codon was mostly prevalent, with only the gene *rps*3 using the alternative “AUU” initiation codon (Deng et al., 2016). In all cases the stop codon was “UAA” (Table 2).

### 3.3. Ribosomal protein, transfer RNA, and ribosomal RNA genes

In *T. koningiopsis* POS7 mitogenome the mitochondrial ribosomal protein S3 (*rps*3) gene was found with a sequence length of 1,401 bp. This gene was also found in the mitogenomes of *T. atroviride* (1,389 bp—MN125601), *T. hamatum* (1,350 bp—MF287973), *T. harzianum* (1,368 bp—MN564945), *T. lixii* (1,386 bp—NC_052832), *T. simmonsii* (1,368 bp—NC_063562), *T. cornu-damae* (1,428 bp—MW525445), and *T. afroharzianum* (1,383 bp—NC_065768). Furthermore, in all of these mitogenomes, except *T. cornu-damae,* the *rps*3 gene is located into the large subunit ribosomal RNA (LSU rRNA; Supplementary Table S1). The *rps*3 gene of *T. koningiopsis* had elevated identity (> 91%) with the other *rps3* gene of the *Trichoderma* genus, specifically with that belonging to *T. atroviride* (98.07%).

Across the fungi, the *rps*3 gene is probably implicated in the assembly of the mitochondrial ribosomal subunit and is extremely diverse in location and organization (Korovesi et al., 2018). Some versions of this gene have been incorporated into a group I intron, others appear to have gained large insertions, microsatellite expansions, or have been invaded by homing endonucleases (Sethuraman et al., 2009; Wai et al., 2019). Among these ones, the *rps*3 gene of *T. afroharzianum* is located into the LSU rRNA, together the LAGLIDADG, one of the two types of homing endonucleases known in fungal mitogenomes (Megarioti and Kouvelis, 2020), and the *rps*3 gene of *T. lixii* is located into an intron IA with 1,634 bp (Supplementary Table S1).

Also, *rps*3 gene, that is most likely native to mitogenome, can be lost, likely due to the presences of a nuclear-encoded analog or homolog that could complement for a missing mitochondrial-encoded *rps*3 gene (Wai et al., 2019). Analyzing mitogenomes from *Trichoderma* representatives, the *rps*3 gene seems have been in *T. gamsii* (NC_030218), *T. pseudokoningii* (OW971927) and *T. asperellum* (NC_037075) but was lost. These events may be resulted of a remnant of a mechanism that led to Organellar Gene Transfer (OGT) of the mt genes to the nucleus or to extinction, because the *rps*3 gene is an ancient gene whose evolutionary history may reflect the evolution of the fungal mitogenomes (Korovesi et al., 2018). Interestingly, in *T. virens* (1,411 bp, CP071122) and *T. reesei* (1,425 bp, NC_003388) a *rps*5 gene with similar length to the *rps*3 gene is found. Furthermore, this *rps*5 gene in the latter two mitogenomes is located into the LSU rRNA too (Supplementary Table S1).

**Figure 2:**
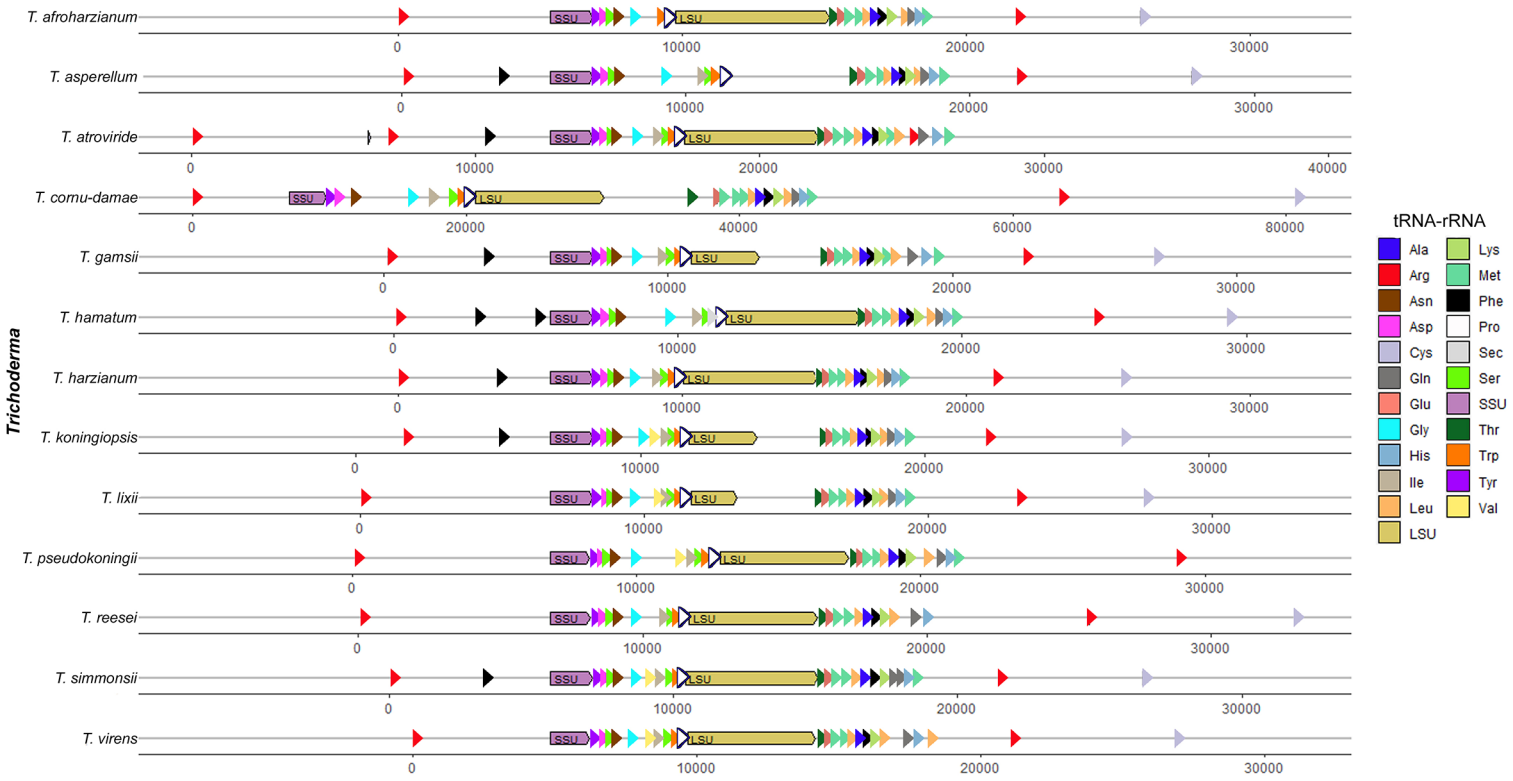
Annotated tRNA and rRNA genes order from analyzed *Trichoderma* mitogenomes. All tRNA and rRNA genes in their mitogenomes were arbitrarily organized starting immediately after the *cox*1 gene.

A total of 26 tRNA genes were identified on *T. koningiopsis* POS7 mitogenome using tRNAscan. Among the tRNAs, all the 20 for standard amino-acids were accounted for, but for some amino-acids multiple tRNAs were found. There were found two copies of the tRNAs genes for arginine, phenylalanine, serine, and leucine, and three copies of the tRNA gene for methionine. No introns have been found in the tRNA genes detected. The number of tRNA genes reported in the 12 other *Trichoderma* mitogenomes ranged from 22 in *T. afroharzianum* (NC_065768) to 27 in *T. atroviride* (MN125601), and the tRNA genes with more than one copy, in general, are the same of the reported in *T. koningiopsis* POS7 mitogenome (Table 3; Figure 2).

tRNA genes are an important nexus between mRNAs and proteins, as they are a fundamental component of the translation machinery in that they deliver amino acids to the ribosome to translate the genetic information in a mRNA (Kirchner and Ignatova, 2015). Mitochondrial tRNA mutations have been demonstrated to be associated with metabolism and various diseases in humans; however, little research has been done on tRNA mutations in fungal mitogenomes related to the growth and pathogenicity of pathogenic fungi (Chen et al., 2019).

It was reported a characteristic clustering of the *trn* genes in the mtDNA to all *Pezizomycotina*, just with minor differences, especially the cluster of 12 *trn* genes, located between *rnl* and *nad*2 genes in all *Sordariomycetes* remained almost unchanged both in gene content and order (Ghikas et al., 2006; Aguileta et al., 2014). In *T. koningiopsis* mitogenome, this cluster of 12 *trn* genes was identical to the cluster reported for *T. pseudokoningii* mitogenome, and it only had one less *trn*M gene between the third and the fourth *trn* genes compared to the cluster found in the *T. lixii* (NC_052832) and *T. hamatum* (MF287973) mitogenomes (Supplementary Table S1; Figure 2).

Two mitochondrial rRNA genes were identified in the *T. koningiopsis* POS7 mitogenome, namely the large subunit ribosomal RNA (*rnl*) and the small subunit ribosomal RNA (*rns*) genes. These genes were similar to the others compared *Trichoderma* fungal mitogenomes (Table 3; Figure 2), with one exception, in the case of *T. asperellum* (NC_037075) which had only one mitochondrial rRNA gene reported (Kwak, 2020). The *rnl* gene in the *T. koningiopsis* POS7 mitogenome was 4,702 bp in length with an intronic sequence of 1,750 bp containing the *rps*3 gene. The *rns* gene was a complete gene sequence of 1,503 bp without any intronic spacer identified. The *rns* genes of *T. reesei* (NC_003388), *T. virens* (CP071122), *T. afroharzianum* (NC_065768), *T. pseudokoningii* (OW971927) and *T. cornu-damae* (MW525445) had a similar intronic sequence (Table 3; Supplementary Table S1). Furthermore, the *rnl* gene of *T. koningiopsis* had a high identity (> 94%) with the other *rns* genes of the *Trichoderma* genus, particularly this gene had the highest identity (98.41%) with the *rnl* gene of *T. atroviride*.

### 3.4. Intronic and intergenic regions

Mitochondrial introns are divided into two groups (I and II) based on their secondary structure and splicing mechanism (Zhang and Zhang, 2019). Group I introns are considered to be mobile genetic elements which interrupt protein-coding and RNA genes in all domains of life (Edgell et al., 2011). In fungi, introns are inserted in many different mitochondrial genes, with a strong preference for protein-coding genes, most frequently *cox* and *cob* (Paquin et al., 1997; Megarioti and Kouvelis, 2020). However, despite the small genome size, two introns were detected in the *T. koningiopsis* POS7 mitogenome.

The intron of 274 bp in *cox*3 gene had a high identity (BLAST) with the intron 1 in *cox*3 gene of *Cordyceps militaris* (OM203116 and MW387534, 96.3% 1*e*–40) and *Cordyceps cicadae* (NC_041489, MK110677, and MH922223, 91.59% 4*e*–31), which are an intron Group IB. In addition, the another intron found in *T. koningiopsis* POS7 mitogenome had a size of 1,750 bp was detected in the *rnl* gene. The latter intron had high identity (BLAST; 97.6%, 1e-0) with the mitogenomes of *T. atroviride* (NC_048477 and MN125601), *T. gamsii* (NC_030218 and KU687109) and *T. hamatum* (MF287973 and NC_036144).

Related to the other species in the genus, *T. asperellum* (NC_037075) has one intron of 1,118 bp into *cox*3 gene reported in its mitogenome (3.73%), and *T. hamatum* (MF287973) has four introns reported with a total size of 4,825 bp (14.73%), one of these is located into *cox*3 gene with a total size of 1,112 bp. Related to introns into *rnl* gene, *T. reesei* (NC_003388) has nine introns reported with a total size of 10,963 bp (26.02%), and one of these is located into *rnl* gene with a total size of 1,535 bp. *T. virens* (CP071122) has three introns reported with a total size of 2,720 bp (8.75%), and one of these is located also into *rnl* gene with a total size of 1,520 bp. *T. pseudokoningii* (OW971927) has eight introns reported with a total size of 8,805 bp (19.52%), and one of these is located into *rnl* gene with a total size of 1,539 bp, and the mitogenomes of *T. afroharzianum* (NC_065768, total size of 4,000 bp—13.55%) and *T. cornu-damae* (MW525445, total size of 45,522 bp—48.12%) have many identified introns into their genes, and particularly have two and four introns into their *rnl* genes, respectively (Table 3). So, the existence of only two intronic sequences in *T. koningiopsis* POS7 mitogenome might constitute an intermediate condition in the continuum reported for the representatives of *Trichoderma* genus, accounting for 7.34% of this mitogenome with a total size of 2,024 bp.

The high evolution rate of mitogenomes makes that amount and localization of their introns can lead to explain variability found among different strains within the same genus or even within a species (Deng et al., 2016). Related to introns, one evolutive hypothesis suggests that within any particular host lineage, a dynamic cycle of invasion of them might be the result of several processes, including horizontal transmission, degeneration or eventual loss followed by reinvasion (Goddard and Burt, 1999; Mardanov et al., 2014). According to other authors, intron variation can be explained evolutionarily using theories proposed for all organisms. One theory, called “Early Intron,” proposes that introns were abundant in ancestral genes, but a general evolutionary process led to the loss of introns over time (Goddard and Burt, 1999; Gonzalez et al., 1999). Another theory, known as the “Late Intron,” suggests that intron mobility allows for expansion within genes due to events of horizontal transfer, even between phylogenetically distant species (Vaughn et al., 1995; Gonzalez et al., 1998). A third hypothesis called “aenaon” combines features of the first two models (Megarioti and Kouvelis, 2020). On the basis of the number of introns, sequence identities, and the locations of them in related species, we could hypothesize that the variation of introns in *T. koningiopsis* was related to this third theory.

All this information could suggests that the number of introns in mitogenomes might contribute to the variability such as it was inferred here where *T. cornu-damae* (94,608 bp—MW525445) had the largest mitogenome with 48.12% of introns, versus the shortest mitogenome of *T. koningiopsis* with 7.34% (Hausner, 2003; Deng et al., 2016; Kanzi et al., 2016; Fan et al., 2019; Lee et al., 2022). However, on the basis of available studies about mitochondrial gene order, Aguileta et al. (2014) did not found a significant correlation between rearrangements of the fungal mitogenomes and the proportions of introns and intronic ORF.

Other authors indicated that the length of intergenic regions is also other key contributor to mitochondrial genomic size variation in fungi (Lin et al., 2015). In the *T. koningiopsis* POS7 mitogenome, the smallest mitogenome in the genus, the intergenic regions accounted for a total of 5,960 bp (21.63% of the total mitgenome), with lengths ranging from 4 to 890 bp. The largest intergenic region was between *nad*5 and *cob* genes. Meanwhile, in all of the other analyzed mitogenomes, the length of intergenic regions was similar to *T. koningiopsis*, with the exception of the species with the largest mitogenome in the genus *T. cornu-damae* (MW525445; Kwak, 2020; Lee et al., 2022). So, in *Trichoderma*, intergenic regions are not proposed as a key force modeling mitochondrial genomic size variation.

### 3.5. Phylogenetic analyses

The most common approach for phylogenetic studies of fungi with mitochondrial sequences is using a set of conserved proteins from their annotated sequences. Since mitogenomes corresponding to the evaluated species are considered to be circular molecules, the alignment start position was arbitrarily decided as the *cox*1 gene (Jelen et al., 2016).

**Figure 3:**
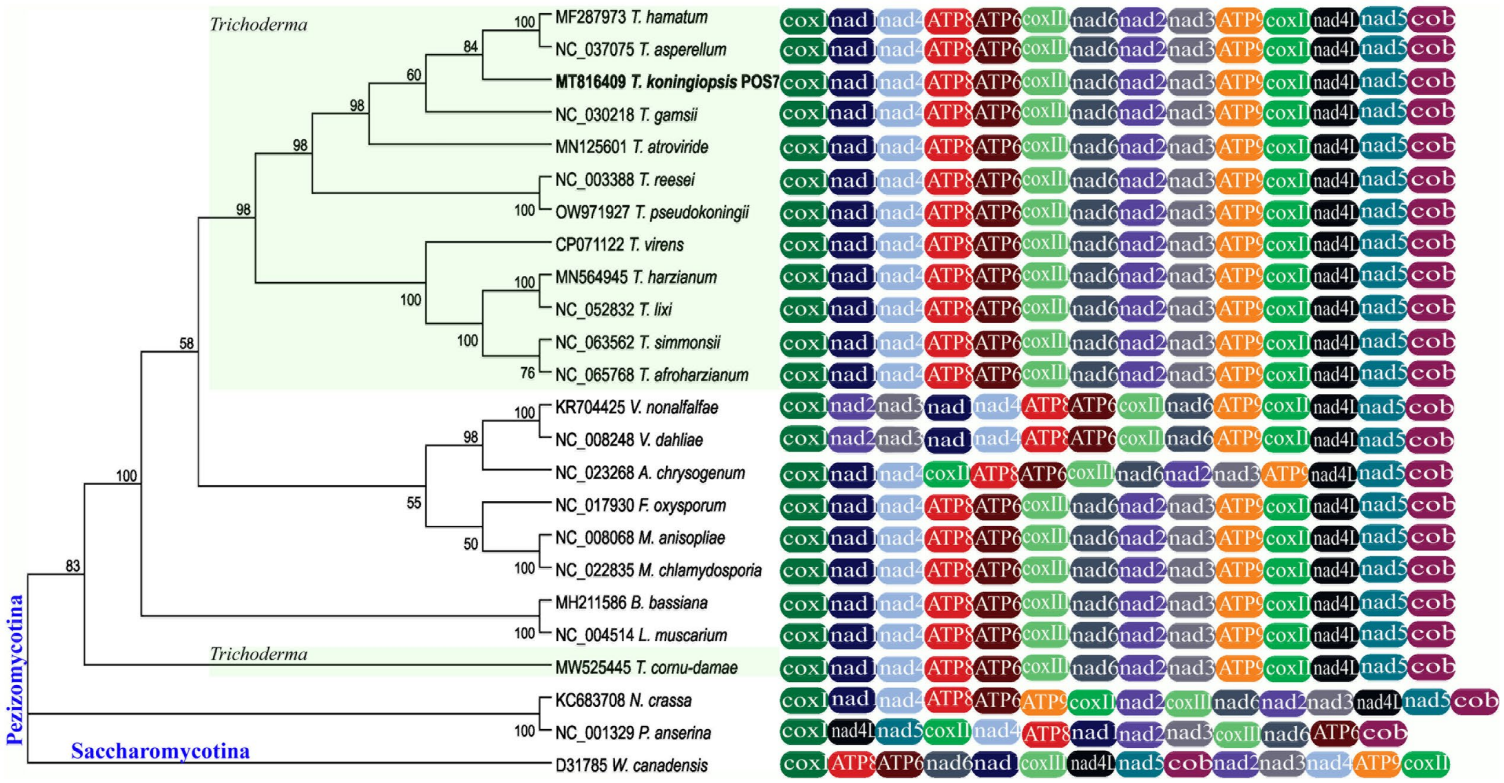
The *Trichoderma koningiopsis* POS7 phylogenetic analysis of 14 mitochondrial protein-encoding genes through ML. One species from the Saccharomycotina subphylum (*Wickerhamomyces Canadensis* D31785) were used as outgroup. The mtDNA *T. koningiopsis* POS7 is indicated in bold letters. Numbers on branches correspond to bootstrap values obtained with 1,000 replicates. Nodes with Bootstraps values below 50% were collapsed.

Mitogenomes of *T. koningiopsis* POS7 and of 23 other selected fungi (Table 1) were used to construct ML and NJ phylogenetic trees. Both algorithms yielded single trees with the same branch topology (for clarity only the NJ tree is presented in Figure 3).

Based on the phylogenetic analysis of the 14 conserved genes used, the 24 Ascomycota species could be divided into two major clades corresponding to the subphyla Pezizomycotina and Saccharomycotina (this latter used as outgroup). Most nodes in this tree had high bootstrap values which indicate the robustness of the phylogenetic tree inferred.

In the Pezizomycotina clade, mitogenomes of the orders Hypocreales and Glomerellales were clustered including a big clade containing all *Trichoderma* mitogenomes (support value 98%), with the exception of the largest *Trichoderma* mitogenome, *T. cornu-damae* (MW525445).

A similar pattern of phylogeny branching was obtained for fungi of Ascomycota by Ghikas et al. (2006), for selected Basidiomycota and Ascomycota by Aguileta et al. (2014), for Pezizomycotina by Cardona et al. (2018), and for Hypocreales by Wang et al. (2017) and Lee et al. (2022). Therefore, the combination of 14 mitochondrial gene set analyzed in this work may be a useful strategy for some fungal taxa identification and phylogenetic analysis.

Even it has been reported that the mitochondrial gene order in fungi varied greatly among different species, even among strains from the same species (Li et al., 2019). In the present study, the mitochondrial gene order of *T. koningiopsis* was identical to all other *Trichoderma* mitogenomes reported (Supplementary Table S1), so we did not find that a gene rearrangement occurred among *Trichoderma* species including other closely related fungal genus involving mitochondrial core genes encoding proteins.

Related to the gene order comparison, 18 of the 19 compared species of the order Hypocreales analyzed have complete synteny in gene order. In contrast, the gene order in the mitogenome of *Acremonium chrysogenum* differs only in the location of the gene *cox*2, between *nad*4 and *atp*8 genes. Lin et al. (2015) evaluated the mitochondrial gene order of different members belonging to Hypocreales and obtained similar results. Also, it was observed that *V. nonalfalfae* and *V. dahliae*, from the Glomerellales order, were clustered together with our evaluated sequences of the Hypocreales order, but it was observed that the *nad*2 and *nad*3 genes shifted in their gene order between the *cox*1 and *nad*1 genes.

Even the fungal mitogenome organization is apparently quite diverse; certain features appear to be conserved in the mitogenome organization of some related fungi like the overlapping of *nad*4L/*nad*5 so that the last base of the first gene was also the first base of the second gene, a feature that is underlined as common in mitogenomes of Sordariomycetes (Kouvelis et al., 2004). It was noted that in general the *nad*4L/*nad*5 and *nad*2/*nad*3 genes, tend to be next to each other, and that in these two pairs of genes existed an overlap of the stop and initiation nucleotides (Aguileta et al., 2014).

Both mitochondrial and nuclear gene sequences have been employed in efforts to reconstruct phylogenetic relationships among fungi. Also, similar phylogeny branching could be obtained using ITS genes for the same *Trichoderma* species (Castrillo, 2015; Castrillo et al., 2021b). But in other groups of organisms the mitochondrial data alone have less resolving power that nuclear genes where in contrast phylogenies from nuclear data is generally well-supported (Springer et al., 2001; Wiens et al., 2010).

Mitochondrial sequences have been found to be useful to determine and/or confirm phylogenetic relationships, especially in cases where nuclear genes are not reliable molecular timers to clarify conflicting phylogenies (Ghikas et al., 2010; Kanzi et al., 2016). Aguileta et al. (2014) argued that although mitochondrial genes tend to be conserved due to their universal role in cellular metabolism, fungal mitochondrial gene order is relatively free to vary, and that this variation is probably largely due to recombination. It is worth mentioning that it was reported that mitochondrial intergenic regions can serve as information for phylogenetic analysis and for the development of tools for intra-and inter-specific discrimination of fungi (Ghikas et al., 2006, 2010).

Other sources of fungal mitogenome variation are presence of repeats, variable numbers of ORFs with an unknown function and variability in the mitochondrial gene order (Jelen et al., 2016). The arrangement of mitochondrial genes has been widely employed to understand the phylogenetic status of species (Ghikas et al., 2006; Li et al., 2019; Chen et al., 2019).

In many cases, it has been shown that a single gene may not always represent the history of the genome containing it, and analyses based on this single gene may lead to wrong conclusions about the phylogenetic relationship of a fungus (Franco et al., 2016). The independent nature of mitochondrial genomes from nuclear genomes presents a promising and alternative source of data for phylogenetic analysis. Thus, the sequence of the entire mitogenome, the set of their 14 conserved protein coding-genes, the set of their tRNA and rRNA sequences, or even only their intergenic sequences, may be considered a promising alternative for phylogenetic analyses. All this can provide an approach to evaluate genetic divergences in related taxons, or offer a thorough view of the placement and types of various genes, as well as any connected introns that may reveal ancestral linkages to study the origin and evolution of eukaryotes, as in the case of fungi (Chen et al., 2019). This might be the case also for inferring phylogenetic uncertainties found in the Hypocreales group. In the last years different *Trichoderma* mitochondrial genome were reported, and 12 were included and analyzed. Therefore, the mitochondrial genome of *T. koningiopsis* POS7 will allow further investigations into evolutionary biology of this important genus as well as other closely related species. Since Medina et al. (2020) reported that both conservative mitochondrial genes and highly variable regions in the fungal mitogenomes are involved in the phenotypic plasticity of hosts, future studies analyzing other isolates of *T. koningiopsis* that differ in their mycoparasitic ability might give information about the contribution of the mitogenome to the behavior of these strains in biocontrol of phytopathogens and for the selection for other biotechnological uses.

## Data availability statement

The datasets presented in this study can be found in online repositories. The names of the repository/repositories and accession number(s) can be found at: https://www.ncbi.nlm.nih.gov/genbank/, MT816499.1.

## Author contributions

MC, GB, NA, MB, PZ, MS, and LV participated in the design of the study. MC, GB, NA, and MB performed the experiments. MC and GB analyzed the data and wrote the paper. All authors contributed to the critical revision of the manuscript and have seen and approved the final draft. All authors read and approved the final manuscript.

## Conflict of interest

The authors declare that the research was conducted in the absence of any commercial or financial relationships that could be construed as a potential conflict of interest.

## Publisher’s note

All claims expressed in this article are solely those of the authors and do not necessarily represent those of their affiliated organizations, or those of the publisher, the editors and the reviewers. Any product that may be evaluated in this article, or claim that may be made by its manufacturer, is not guaranteed or endorsed by the publisher.

## Footnotes

^1^https://www.ncbi.nlm.nih.gov/orffinder/

^2^https://megasun.bch.umontreal.ca/cgi-bin/dev_mfa/mfannotInterface.pl
